# Computing Bayes factors for evidence-accumulation models using Warp-III bridge sampling

**DOI:** 10.3758/s13428-019-01290-6

**Published:** 2019-11-21

**Authors:** Quentin F. Gronau, Andrew Heathcote, Dora Matzke

**Affiliations:** 1 University of Amsterdam, Amsterdam, Netherlands; 2 University of Tasmania, Hobart, Australia

**Keywords:** Bayesian model comparison, Differential evolution Markov chain Monte Carlo, Dynamic models of choice, Linear ballistic accumulator, Marginal likelihood, Response time models

## Abstract

Over the last decade, the Bayesian estimation of evidence-accumulation models has gained popularity, largely due to the advantages afforded by the Bayesian hierarchical framework. Despite recent advances in the Bayesian estimation of evidence-accumulation models, model comparison continues to rely on suboptimal procedures, such as posterior parameter inference and model selection criteria known to favor overly complex models. In this paper, we advocate model comparison for evidence-accumulation models based on the Bayes factor obtained via Warp-III bridge sampling. We demonstrate, using the linear ballistic accumulator (LBA), that Warp-III sampling provides a powerful and flexible approach that can be applied to both nested and non-nested model comparisons, even in complex and high-dimensional hierarchical instantiations of the LBA. We provide an easy-to-use software implementation of the Warp-III sampler and outline a series of recommendations aimed at facilitating the use of Warp-III sampling in practical applications.

## Introduction

Cognitive models of response times and accuracy canonically assume an accumulation process, where evidence favoring different options is summed over time until a threshold is reached that triggers an associated response. The two most prominent types of evidence-accumulation models, the diffusion decision model (DDM; Ratcliff, 1978; Ratcliff & McKoon, 2008) and the linear ballistic accumulator (LBA; Brown & Heathcote, 2008) have been widely applied across animal and human research in biology, psychology, economics, and the neurosciences to topics including vision, attention, language, memory, cognition, emotion, development, aging, and clinical disorders (for reviews, see Mulder, Van Maanen, & Forstmann, 2014; Ratcliff, Smith, Brown, & McKoon, 2016; Donkin & Brown, 2018). Evidence-accumulation models are popular because they provide a comprehensive account of the probability of choices and the associated distribution of times to make them, and because they provide parameter estimates that directly quantify important psychological quantities, such as the quality of the evidence provided by a choice stimulus and the amount of evidence required to trigger the response.

Parameter estimation and statistical inference in the context of evidence-accumulation models can be challenging because they belong to the class of “sloppy” models with highly correlated parameters (Apgar, Witmer, White, & Tidor, 2010; Gutenkunst et al.,, 2007), examples of which occur widely in biology and psychology (Apgar et al., 2010; Gutenkunst et al., 2007; Heathcote et al., 2018). However, with appropriate experimental designs—critically including sufficiently high error rates and experimental trials per participant (Ratcliff & Childers, 2015)—the model parameters can be estimated reliably using error minimization and Bayesian methods.

Recently, the Bayesian estimation of evidence-accumulation models has gained popularity, largely due to the advantages afforded by the Bayesian hierarchical framework (e.g., Heathcote et al.,, 2018; Vandekerckhove, Tuerlinckx, & Lee, 2011; Wiecki, Sofer, & Frank, 2013). In fact, our recent literature review indicated that 19% and 21% of the 262 and 53 papers that used the DDM and the LBA, respectively, relied on Bayesian methods to estimate the model parameters.^1^ Bayesian hierarchical methods simultaneously estimate model parameters for a group of participants assuming that the participant-level parameters are drawn from a common group-level distribution. From a statistical point of view, the group-level distribution acts as a prior that pulls (“shrinks”) the participant-level parameters to the group mean, which can result in less variable and, on average, more accurate estimates than non-hierarchical methods (Farrell & Ludwig, 2008; Gelman & Hill, 2007; Lee & Wagenmakers, 2013; Shiffrin, Lee, Kim, & Wagenmakers, 2008). From a psychological point of view, the group-level distribution provides a model of individual differences. From this perspective, it is apparent that introducing a group-level distribution improves the model theoretically only if the group-level distribution provides a good model for the individual variation (Farrell & Lewandowsky, 2018, section 9.5).

As a result of the strong parameter correlations in evidence-accumulation models, standard Markov chain Monte Carlo samplers (MCMC; e.g., Gilks, Richardson, & Spiegelhalter, 1996) typically used for Bayesian parameter estimation can be inefficient. Rather, samplers designed to handle high posterior correlations must be used, such as differential evolution MCMC (DE-MCMC; Turner, Sederberg, Brown, & Steyvers, 2013). This approach to Bayesian estimation is now readily available for the DDM, LBA, and other evidence-accumulation models in the “Dynamic Models of Choice” software (DMC; Heathcote et al.,, 2018) along with extensive tutorials and supporting functions that facilitate model diagnostics and the analysis of results.^2^ In this article, we focus on the Bayesian approach because of the advantages it offers, such as a coherent inferential framework, the use of prior information, the possibility of straightforward hierarchical extensions, and the natural quantification of uncertainty in both parameter estimates and model predictions.

In typical applications of evidence-accumulation models, researchers are not only interested in parameter estimation but often wish to assess the effects of experimental manipulations on the model parameters. For example, Strickland, Loft, Remington, and Heathcote (2018) compared non-nested LBA models that either allowed the effect of maintaining a prospective memory load (i.e., in the context of a routine ongoing task, the intent to make an alternative response to a rarely occurring stimulus) to influence only the rate of evidence accumulation or only the threshold amount of evidence required to make a response. The former model corresponds to competition for limited information-processing capacity, whereas the latter model corresponds to strategic slowing in order to avoid the ongoing task response pre-empting the prospective memory response (Heathcote, Loft, & Remington, 2015). Nested comparisons are also common in the context of evidence-accumulation models to determine which of a set of candidate experimental manipulations had an effect on a particular parameter. For example, Rae, Heathcote, Donkin, Averell, and Brown (2014) examined whether or not an emphasis on the speed vs. accuracy of responding influences evidence accumulation rates.

Despite recent advances in the Bayesian estimation of evidence-accumulation models, model comparison continues to rely on suboptimal procedures, such as posterior parameter inference based on complex models where separate model parameters are estimated for each experimental condition. In this approach, differences between parameters are often evaluated using posterior *p* values (e.g., Klauer, 2010; Matzke, Dolan, Batchelder, & Wagenmakers, 2015; Matzke, Hughes, Badcock, Michie, & Heathcote, 2017; Matzke, Boehm, & Vandekerckhove, 2018; Smith & Batchelder, 2010; Strickland et al.,, 2018; Tilman, Osth, van Ravenzwaaij, & Heathcote, 2017; Tilman, Strayer, Eidels, & Heathcote, 2017; Osth, Jansson, Dennis, & Heathcote, 2018). Posterior parameter inference has at least three limitations. First, it can only be used for nested model comparison. Second, it cannot provide evidence for the absence of an effect (i.e., it cannot “prove the null”), similar to classical *p* values (e.g., Wagenmakers, 2007). Third, it can result in fitting an overly complex model, which is particularly problematic in the presence of strong parameter correlations, because a real effect in one parameter can spread to create a spurious effect on other parameters (Heathcote et al., 2015).

These shortcomings can be addressed using formal model selection. This approach critically depends on the availability of a model selection criterion that properly penalizes the greater flexibility of more complex models. The deviance information criterion (DIC) is one of the most commonly used model selection measures, and has the advantage that it can be easily computed from the posterior samples obtained during parameter estimation. However, the DIC is known to prefer overly complex models (Spiegelhalter, Best, Carlin, & van der Linde, 2002). The more recent widely applicable information criterion (WAIC; Vehtari, Gelman, & Gabry, 2017), which is also based on posterior samples, is an approximation to (leave-one-out) cross-validation and suffers from the same shortcoming (Browne, 2000). It should be noted that even as the number of observations goes to infinity, methods that approximate (leave-one-out) cross-validation will not choose the data-generating model with certainty (Shao, 1993).

Here we advocate model selection for evidence-accumulation models based on the *Bayes factor* (e.g., Etz & Wagenmakers, 2017; Kass & Raftery, 1995; Ly, Verhagen, & Wagenmakers, 2016; Jeffreys, 1961). The Bayes factor is the principled method of performing model selection from a Bayesian perspective and follows immediately from applying Bayes’ rule to models instead of parameters (e.g., Kass & Raftery, 1995). In contrast to model selection methods that approximate (leave-one-out) cross-validation, in general, the Bayes factor will choose the data-generating model with certainty when the number of observations goes to infinity (Bayarri, Berger, Forte, & García-Donato, 2012). Although the desirability of Bayes factors has long been recognized (e.g., Jeffreys, 1939), their use has only become increasingly widespread with general linear models (e.g., ANOVA and regression; see Rouder, Morey, Speckman, & Province, 2012 and Rouder & Morey, 2012) due to the availability of efficient and user-friendly software implementations in packages such as BayesFactor (Morey & Rouder, 2018) in R (R Core Team, 2019) and the GUI-based JASP (JASP Team, 2018). With this article, we aim to bring these advantages to the domain of evidence-accumulation models by providing an easy-to-use software implementation that uses a state-of-the-art method for computing Bayes factors.

The Bayes factor is the predictive updating factor that changes prior model odds for two models ${\mathscr{M}}_{1}$ and ${\mathscr{M}}_{2}$ into posterior model odds based on observed data ***y***:

1 $$  \underbrace{\frac{p(\mathcal{M}_{1} \mid \boldsymbol{y})}{p(\mathcal{M}_{2} \mid \boldsymbol{y})}}_{\text{posterior odds}} = \underbrace{\frac{p(\boldsymbol{y} \mid \mathcal{M}_{1})}{p(\boldsymbol{y} \mid \mathcal{M}_{2})}}_{\text{Bayes factor BF$_{12}$}} \times   \underbrace{\frac{p(\mathcal{M}_{1})}{p(\mathcal{M}_{2})}}_{\text{prior odds}}. $$

Continuing the example from Strickland et al., (2018), suppose that ${\mathscr{M}}_{1}$ refers to the model in which only rates are affected by prospective-memory load and ${\mathscr{M}}_{2}$ refers to the model in which only thresholds are affected. Different researchers may start with different prior beliefs about the relative plausibility of the two competing psychological explanations of the prospective-memory load effect. However, the change in beliefs brought about by the data (i.e., the change from prior to posterior odds which is the Bayes factor) is the same, regardless of the prior beliefs. Therefore, reporting the Bayes factor enables researchers to update their personal prior odds to posterior odds. Commonly, only the Bayes factor is reported and interpreted, since strength of evidence for the two competing models is naturally expressed as the degree to which one should update prior beliefs about the models based on observed data. A Bayes factor of, say, BF_12_ = 10 would indicate that the data are ten times more likely under ${\mathscr{M}}_{1}$ than ${\mathscr{M}}_{2}$, whereas a Bayes factor of BF_12_ = 0.1 would indicate that the data are ten times more likely under ${\mathscr{M}}_{2}$ than ${\mathscr{M}}_{1}$.

As shown in Eq. 1, the Bayes factor is the ratio of the *marginal likelihoods* of the models. The marginal likelihood is the probability of the data given a model and is obtained by integrating out the model parameters with respect to the parameters’ prior distribution:

2 $$  p(\boldsymbol{y} \mid \mathcal{M}) = {\int}_{\boldsymbol{\Theta}} p(\boldsymbol{y} \mid \boldsymbol{\theta}, \mathcal{M}) \thinspace p(\boldsymbol{\theta} \mid \mathcal{M}) \text{d}\boldsymbol{\theta}, $$

where ***𝜃*** denotes the parameter vector for model ${\mathscr{M}}$. The marginal likelihood quantifies average predictive adequacy as follows: The likelihood $p(\boldsymbol {y} \mid \boldsymbol {\theta }, {\mathscr{M}})$ corresponds to the predictive adequacy of a particular parameter setting ***𝜃*** under model ${\mathscr{M}}$. The average predictive adequacy (i.e., the marginal likelihood) is obtained as the weighted average of the predictive adequacies across the entire parameter space, where the weights are given by the parameters’ prior probabilities. Complex models may have certain parameter settings that yield high likelihood values, however, the large parameter space may also contain many parameter settings which result in small likelihood values, lowering the weighted average. Consequently, the marginal likelihood—and the Bayes factor, which contrasts the average predictive adequacy of two models—incorporates a natural penalty for undue complexity. Interpreting the marginal likelihood as a weighted average highlights the crucial importance of the prior distribution for Bayesian model comparison.

For evidence-accumulation models, the integral in Eq. 2—and hence the Bayes factor—cannot be computed analytically. In these cases, four major approaches are available for computing Bayes factors: (1) approximate methods such as the Laplace approximation (e.g., Kass and Vaidyanathan, 1992); (2) the Savage–Dickey density ratio approximation of the Bayes factor (Dickey & Lientz, 1970; Wagenmakers, Lodewyckx, Kuriyal, & Grasman, 2010); (3) transdimensional methods such as reversible jump MCMC (Green, 1995); and (4) simulation-based methods that estimate the integrals involved in the computation of the Bayes factor directly (e.g., Evans & Brown, 2018; Evans & Annis, 2019; Meng & Wong, 1996; Meng & Schilling, 2002). Approximate methods have the disadvantage that it is typically difficult to assess the approximation error, which could be particularly substantial for hierarchical evidence-accumulation models. The Savage–Dickey density ratio can only be applied to nested model comparisons. Transdimensional methods are challenging to implement, especially in hierarchical settings and for non-nested model comparisons, as explained in more detail later.

Therefore, here we advocate *Warp-III bridge sampling* (Meng & Schilling, 2002) for obtaining the Bayes factor for evidence-accumulation models. Warp-III bridge sampling is a simulation-based method that can be applied to both nested and non-nested comparisons and—once posterior samples from the competing models have been obtained—it is straightforward to implement even in hierarchical settings. As non-nested hierarchical comparisons are integral to many applications of cognitive models, we believe that Warp-III bridge sampling provides an excellent computational tool that will greatly facilitate the use of Bayesian model comparison for evidence-accumulation models.

The article is organized as follows. First, we review simple Monte Carlo sampling, another simulation-based method that has been proposed for computing the Bayes factor for evidence-accumulation models. We then outline the details of Warp-III bridge sampling and illustrate its use for the single-participant as well as the hierarchical case. We focus on the LBA, but elaborate on the applicability of our approach to other evidence-accumulation models, for instance the DDM, in the Discussion. The Discussion also provides recommendations aimed at facilitating the use of Warp-III bridge sampling in practical applications. The implementation of the Warp-III bridge sampler is available at https://osf.io/ynwpa/ and has also been incorporated into the latest DMC release.^3^

## Simple Monte Carlo sampling

A simple Monte Carlo estimator of the marginal likelihood is obtained by interpreting the integral in Eq. 2 as an expected value with respect to the parameters’ prior distribution:

3 $$ \begin{array}{@{}rcl@{}} p(\boldsymbol{y} \mid \mathcal{M})& = & \mathbb{E}_{p(\boldsymbol{\theta} \mid \mathcal{M})}\left[p(\boldsymbol{y} \mid \boldsymbol{\theta}, \mathcal{M})\right]\\ & \approx & \frac{1}{N} {\sum}_{i = 1}^{N} p(\boldsymbol{y} \mid \tilde{\boldsymbol{\theta}}_{i}, \mathcal{M}), \text{where}  \tilde{\boldsymbol{\theta}}_{i}  \sim  p(\boldsymbol{\theta} \mid \mathcal{M}). \end{array} $$

Thus, an estimate of the marginal likelihood can be obtained by sampling from the prior distribution and averaging the likelihood values based on the samples.

Recently, Evans and Brown (2018) proposed the use of simple Monte Carlo sampling for the computation of the Bayes factor for the LBA. This simple approach can work well if the posterior distribution is similar to the prior distribution; however, when the posterior is substantially different from the prior—as is often the case—simple Monte Carlo sampling becomes very inefficient. The reason is that only a few prior samples (i.e., those in the region where most posterior mass is located) result in substantial likelihood values so that the average in Eq. 3 will be dominated by a small number of samples. The result is an unstable estimator, even in non-hierarchical applications. Naturally, the problem becomes more severe in hierarchical settings where the parameter space is substantially larger. Although increasing the number of prior samples may remedy the problem to a certain extent, reliable estimation of the marginal likelihood of hierarchical evidence-accumulation models using simple Monte Carlo sampling remains challenging, even with Evans & Brown’s powerful GPU implementation. Given the many advantages of the Bayesian hierarchical framework for cognitive modeling (e.g., Heathcote et al.,, 2018; Shiffrin et al.,, 2008; Matzke et al.,, 2015; Lee, 2011; Matzke, Dolan, Logan, Brown, & Wagenmakers, 2013; Lee & Wagenmakers, 2013; Vandekerckhove et al.,, 2011; Wiecki et al.,, 2013), we believe that an alternative approach is needed.

## Warp-III bridge sampling

We propose the use of Warp-III bridge sampling (Meng & Schilling, 2002, henceforth referred to as *Warp-III*) for estimating the marginal likelihood for evidence-accumulation models. Warp-III is an advanced version of bridge sampling (Meng & Wong, 1996; Gronau et al., 2017), which is based on the following identity:

4 $$  p(\boldsymbol{y} \mid \mathcal{M}) = \frac{\mathbb{E}_{g(\boldsymbol{\theta})}\left[h(\boldsymbol{\theta})  p(\boldsymbol{y} \mid \boldsymbol{\theta}, \mathcal{M})  p(\boldsymbol{\theta} \mid \mathcal{M})\right]}{\mathbb{E}_{p(\boldsymbol{\theta} \mid \boldsymbol{y}, \mathcal{M})}\left[h(\boldsymbol{\theta})  g(\boldsymbol{\theta})\right]}, $$

where *g* is a proposal distribution and *h* a bridge function.

The efficiency of the bridge sampling estimator is governed by the overlap between the proposal and the posterior distribution. A simple approach for obtaining the bridge sampling estimator relies on a multivariate normal proposal distribution that matches the first two moments, the mean vector and covariance matrix, of the posterior distribution (e.g., Gronau et al.,, 2017; Overstall & Forster, 2010). However, this method becomes inefficient when the posterior distribution is skewed. To remedy this problem, Warp-III aims to maximize the overlap by fixing the proposal distribution to a standard multivariate normal distribution^4^ and then “warping” (i.e., manipulating) the posterior so that it matches not only the first two, but also the third moment of the proposal distribution (for details, see Meng & Schilling, 2002, and Gronau, Wagenmakers, Heck, & Matzke, 2019).

**Fig. 1:**
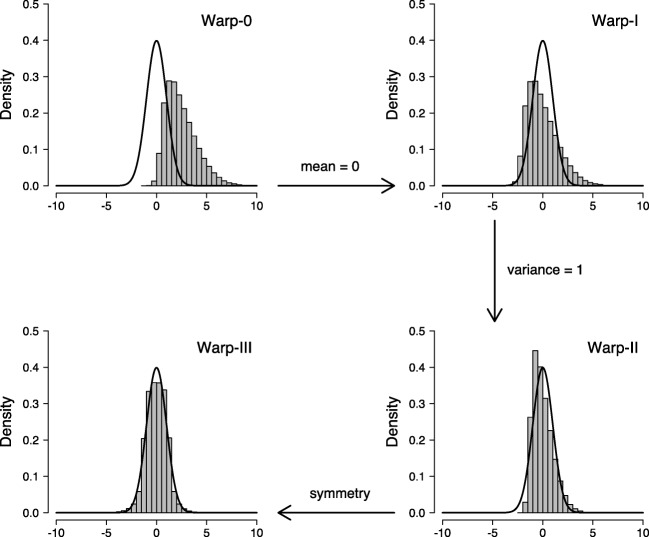
Illustration of the warping procedure. The *solid black line* shows the standard normal proposal distribution and the *gray histogram* shows the posterior samples. Available at https://tinyurl.com/y7owvsz3 under CC license https://creativecommons.org/licenses/by/2.0/

Figure 1 illustrates the warping procedure for the univariate case using hypothetical posterior samples. The solid black line in the top-left panel displays the standard normal proposal distribution and the skewed histogram displays samples from the posterior distribution. Since none of the moments of the two distributions match, applying bridge sampling to these distributions can be called Warp-0 (i.e., the number indicates how many moments have been matched). The histogram in the top-right panel displays the same posterior samples after subtracting their mean from each sample. This manipulation matches the first moment of the two distributions; the posterior samples are now zero-centered, just like the proposal distribution. This is called Warp-I. In the bottom-right panel, the posterior samples are additionally divided by their standard deviation. This manipulation matches the first two moments of the distributions; the posterior samples are now zero-centered with variance 1, just like the proposal distribution. This is called Warp-II. Finally, the bottom-left panel displays the posterior samples after assigning a minus sign with probability 0.5 to each sample. This manipulation achieves symmetry and matches the first three moments of the distributions; the posterior samples are now symmetric and zero-centered with variance 1, just like the proposal distribution. This is called Warp-III. Note how successively matching the moments of the two distributions has increased the overlap between the posterior and the proposal distribution.^5^ We have found that the improvement afforded by Warp-III can be crucial for efficient application of bridge sampling to evidence-accumulation models, particularly in situations where the posteriors are skewed, as is often the case with only a small number of observations per participant.

The bridge function *h* is chosen such that it minimizes the relative mean-square error of the resulting estimator (Meng & Wong, 1996). Using this “optimal” bridge function,^6^ the estimator of the marginal likelihood is obtained by updating an initial guess of the marginal likelihood until convergence. The estimate at iteration *t* + 1 is given by:^7^

5 $$  \hat{p}(\boldsymbol{y} \mid \mathcal{M})^{(t+1)} = \frac{\frac{1}{N_{2}}\sum\limits_{i = 1}^{N_{2}}\frac{l_{2, i}}{s_{1} \thinspace l_{2, i} + s_{2} \thinspace \hat{p}(\boldsymbol{y} \mid \mathcal{M})^{(t)}}}{\frac{1}{N_{1}}\sum\limits_{j = 1}^{N_{1}}\frac{1}{s_{1} \thinspace l_{1, j} + s_{2} \thinspace \hat{p}(\boldsymbol{y} \mid \mathcal{M})^{(t)}}}, $$

where $s_{k} = \frac {N_{k}}{N_{1} + N_{2}}$ for *k* ∈{1,2},

6 $$  l_{1,j} = \tfrac{\frac{\left|\boldsymbol{R}\right|}{2} \left[q(2\boldsymbol{\mu} - \boldsymbol{\theta}^{\ast}_{j}) + q(\boldsymbol{\theta}^{\ast}_{j})\right]}{g\left( \boldsymbol{R}^{-1}\left( \boldsymbol{\theta}^{\ast}_{j} - \boldsymbol{\mu}\right)\right)}, $$

and

7 $$  l_{2,i} =\tfrac{\frac{\left|\boldsymbol{R}\right|}{2} \left[q(\boldsymbol{\mu} - \boldsymbol{R}\tilde{\boldsymbol{\theta}}_{i}) + q(\boldsymbol{\mu} + \boldsymbol{R}\tilde{\boldsymbol{\theta}}_{i})\right]}{g(\tilde{\boldsymbol{\theta}}_{i})}. $$

$\{\boldsymbol {\theta }^{\ast }_{1}, \boldsymbol {\theta }^{\ast }_{2}, \ldots , \boldsymbol {\theta }^{\ast }_{N_{1}}\}$ are *N*_1_ draws from the posterior distribution, $\{\tilde {\boldsymbol {\theta }}_{1}, \tilde {\boldsymbol {\theta }}_{2}, \ldots , \tilde {\boldsymbol {\theta }}_{N_{2}}\}$ are *N*_2_ draws from the standard normal proposal distribution, and $q(\boldsymbol {\theta }) = p(\boldsymbol {y} \mid \boldsymbol {\theta }, {\mathscr{M}}) p(\boldsymbol {\theta } \mid {\mathscr{M}})$ denotes the un-normalized posterior density function. Furthermore, ***μ*** corresponds to the posterior mean vector and **Σ** = ***RR***^⊤^ corresponds to the posterior covariance matrix (***R*** is obtained via a Cholesky decomposition of the posterior covariance matrix). The posterior mean vector and covariance matrix can be estimated using the posterior samples. In practice, we split the posterior samples into two halves; the first half is used to estimate ***μ*** and ***R*** and the second half is used in the iterative scheme in Eq. 5.

Computing *l*_1,*j*_ and *l*_2,*i*_ is the computationally most expensive part of the method; fortunately, these quantities can be computed completely in parallel. Note also that *l*_1,*j*_ and *l*_2,*i*_ only need to be computed once before the updating scheme is started. Hence, with these quantities in hand, running the updating scheme is quick and typically converges in fewer than 20 or 30 iterations. Although our implementation relies on a fixed starting value, it is also possible to start the updating scheme from an informed guess of the marginal likelihood, for instance, based on a normal approximation to the posterior distribution. We have found that the value of the initial guess usually does not influence the resulting estimator substantially, but a good starting value may reduce the number of iterations needed to reach convergence. Moreover, as we show later, an appropriately chosen starting value is crucial in rare cases when the iterative scheme seemingly does not converge.^8^

It can be shown that the simple Monte Carlo estimator described in the previous section is a special case of Eq. 4 obtained by using a bridge function other than the optimal one (e.g., Gronau et al.,, 2017, Appendix A). Therefore, Warp-III that relies on the optimal bridge function must perform better in terms of the relative mean-square error of the estimator than the simple Monte Carlo approach. This will be illustrated in the next section, where we apply Warp-III sampling to a nested model comparison problem and compare its performance to three alternative methods, including simple Monte Carlo sampling.

## Simulation study I: nested model comparison for the single-participant case

As a first example, we computed the Bayes factor for a nested model comparison problem in the LBA by approximating the marginal likelihood of the two models using Warp-III sampling. To verify the correctness of our Warp-III implementation, we also computed the Bayes factor using three alternative methods: (1) simple Monte Carlo sampling; (2) the Savage–Dickey density ratio; and (3) a simple version of reversible jump MCMC (RJMCMC; Green, 1995) as described in Barker and Link (2013). We included the latter two approaches because they provide conceptually different methods for Bayes factor computations than the simulation-based Warp-III and simple Monte Carlo. The details of the Savage–Dickey and the RJMCMC methods are provided in the Appendix.

### Models and data

**Fig. 2:**
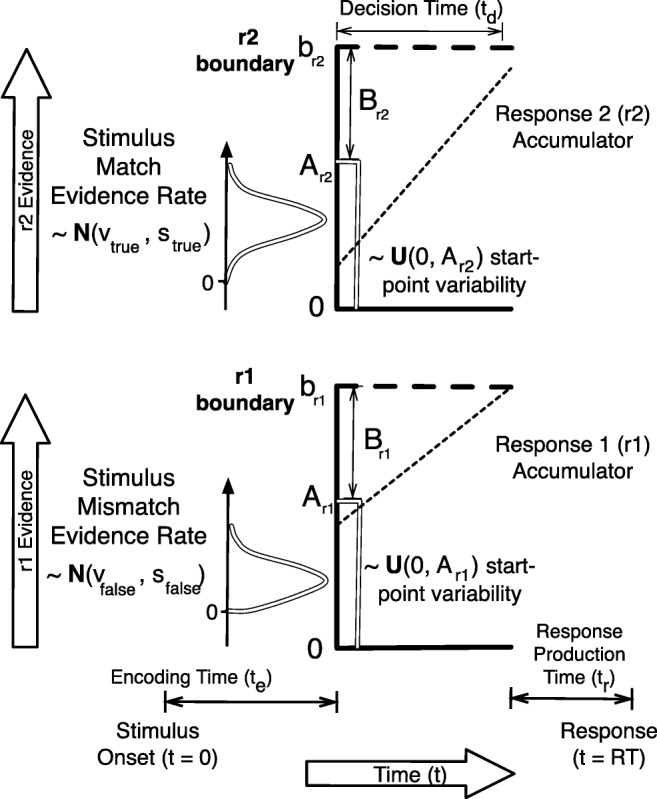
Graphical representation of the linear ballistic accumulator for two possible responses (*r*1 and *r*2) corresponding to two stimuli (*s*1 and *s*2). The figure illustrates a case where *s*2 is presented and the sampled rate for the *r*2 accumulator is greater than the sampled rate for the *r*1 accumulator, i.e., the accumulation path (*dashed line*) is steeper for *r*2 than for *r*1. However, as the sampled starting point for *r*1 is higher than for *r*2, the *r*1 accumulator has a sufficient head start to get to its threshold first after time *t*_*d*_. The resulting response is an error, with *RT* = *t*_0_ + *t*_*d*_. Available at https://tinyurl.com/yc4n8lpm under CC license https://creativecommons.org/licenses/by/2.0/

We considered a data set generated from the LBA for a single participant performing a simple choice task with two stimuli and two corresponding responses. As shown in Fig. 2, the LBA assumes a race among a set of deterministic evidence-accumulation processes, with one runner per response option. The choice is determined by the winner of the race.

On each trial, accumulation begins at a starting point drawn—independently for each accumulator—from a uniform distribution with width *A*. *A* may vary between accumulators, but here we assume it is the same. The evidence total increases linearly at rate *v* that is drawn independently for each accumulator from a normal distribution, which we assume here is truncated below at zero (Heathcote & Love, 2012). The accumulator that matches the stimulus has mean rate *v*_true_ and standard deviation *s*_true_, and the mismatching accumulator *v*_false_ and *s*_false_. In principle, there could be different *v*_true_ and *v*_false_ values for each stimulus, but here we assume they are the same. The first accumulator to reach its threshold (*b*)—again potentially differing between accumulators but assumed to be the same here—triggers the corresponding response. We estimate threshold in terms of a positive quantity, *B*, which quantifies the gap between the threshold and the upper bound of the start-point noise (i.e., *B* = *b* − *A*). Response time (RT) is equal to the time taken to reach threshold plus non-decision time, *t*_0_, which is the sum of the time to initially encode the stimulus and the time to produce a motor response.

We estimated the Bayes factor to compare two nested LBA models. The first, which we refer to as the *full* model, featured a starting point parameter *A*, a threshold parameter *B*, mean drift rate parameters for the matching and mismatching accumulators, *v*_true_ and *v*_false_, and a non-decision time parameter *t*_0_. In order to identify the model, one accumulator parameter must be fixed (Donkin, Brown, & Heathcote, 2009); here we assumed that the standard deviations of the drift rate distributions were fixed to 1. In later simulations, we make only the minimum required assumption of fixing one parameter, in particular assuming *s*_true_ = 1. We generated a data set with 250 trials per stimulus (i.e., a total of 500 trials) from the full model using the following parameter values: *A* = 0.5, *B* = 1, *v*_true_ = 4, *v*_false_ = 3, and *t*_0_ = 0.2.

The full model was compared to a restricted model in which *v*_true_ was fixed to 3.55. The value 3.55 yields a Bayes factor close to one (equivalently, log Bayes factor of zero) and was chosen for two reasons. First, this value facilitates the implementation of the Savage–Dickey density ratio. The Savage–Dickey method relies on estimating the posterior density at the test value, which can be unreliable when the test value falls in the tail of the posterior distribution. We circumvented this problem by using a test value in the restricted model (*v*_true_ = 3.55) relatively close to the generating parameter in the full model (*v*_true_ = 4).

Second, this value makes discriminating between the models difficult, and allows us to point out the difference between inference and model inversion (Lee, 2018). Although the data have been generated from the full model, a Bayes factor close to 1 indicates that the data are just as likely under the restricted model as under the full model. This may at first appear as an undesirable property of the Bayes factor. This reasoning, however, confuses inference and model inversion. Model inversion means that if the data are generated from model ${\mathscr{M}}_{1}$ and one fits the data-generating model ${\mathscr{M}}_{1}$ and an alternative model ${\mathscr{M}}_{2}$, one is able to identify the data-generating model ${\mathscr{M}}_{1}$ based on a model selection measure of interest. Consider, however, the following example. Suppose we are interested in comparing a null model which assumes that there is no difference in non-decision time *t*_0_ between two groups to an alternative model which allows the effect size to be different from zero. Suppose further that the alternative model is the data-generating model and we simulate data for a small number of synthetic participants assuming a small non-zero effect size, resulting in an observed effect size that, for this sample of participants, happens to be approximately zero. As a result, the simpler null model can account for the observed data almost equally well as the more complex data-generating model and may be favored on the ground of parsimony. As more observations are generated from the alternative model, however, it will become clear that the effect size is non-zero, and the support for the simpler null model will decrease—equivalently, the support for the more complex alternative model will increase. Hence, with a large enough number of observations, model inversion may be fulfilled.

This discussion highlights why the Bayes factor for the simulated LBA data set is indifferent: the number of trials is relatively small and the misspecified simpler model fixes *v*_true_ to 3.55, which is close to the data-generating value of 4. Therefore, the slight misspecification of the simpler restricted model is almost perfectly balanced out by its parsimony advantage compared to the more complex full model. The example is meant as a reminder that Bayesian inference conditions on the data at hand and that it may be reasonable to obtain evidence in favor of a different model than the data-generating one for certain data sets. Therefore, although one can assess the predictive adequacy of two competing models for the observed data using the Bayes factor (Wagenmakers et al., 2018), the Bayes factor should not be expected to necessarily recover a data-generating model in a simulation study. Nevertheless, as the number of observations grows large, the Bayes factor should select the correct model, a property known as model selection consistency (Bayarri et al., 2012).

### Prior distributions

We used the following prior distributions for the different parameter types:

8 $$ \begin{array}{@{}rcl@{}} A &\sim& \mathcal{N}_{+}(1, 1) \\ B &\sim& \mathcal{N}_{+}(1, 1) \\ v_{\text{true}} &\sim& \mathcal{N}(2, 3^{2}) \\ v_{\text{false}} &\sim& \mathcal{N}(1, 3^{2}) \\ t_{0} &\sim& \mathcal{N}_{(0.1, \infty)}(0.3, 0.25^{2}), \end{array} $$

where $\mathcal {N}(\mu , \sigma ^{2})$ denotes a normal distribution with mean *μ* and variance *σ*^2^, $\mathcal {N}_{+}(\mu , \sigma ^{2})$ denotes a normal distribution truncated to allow only positive values, and $\mathcal {N}_{(x,y)}(\mu , \sigma ^{2})$ denotes a normal distribution with lower truncation *x* and upper truncation *y*. In the full model, we specified a prior distribution for all parameters, including *v*_true_. In the restricted model, we specified a prior distribution for all parameters except *v*_true_, as *v*_true_ was fixed to 3.55.

The priors in Eq. 8 were taken from Heathcote et al., (2018). Although we believe that these priors provide a reasonable setup based on our experience with the LBA parameter ranges, they may be replaced by empirically informed priors in future applications. We also acknowledge that our prior choices are for many parameters wider than the ones used by Evans and Brown (2018); this may make the simple Monte Carlo method less efficient than when used in combination with the Evans–Brown priors.

### Parameter estimation and model comparison

We used the DE-MCMC algorithm, as implemented in the DMC software (https://osf.io/pbwx8/) to estimate the model parameters. We set the number of MCMC chains to three times the number of model parameters; for the full model we ran 15 and for the restricted model we ran 12 chains with over-dispersed start values. In order to reduce auto-correlation, we thinned each MCMC chain to retain only every 10^*th*^ posterior sample. During the burn-in period, the probability of a migration step was set to 5%; after burn-in, migration was turned off and only crossover steps were performed. Convergence of the MCMC chains was assessed by visual inspection and the $\hat {R}$ statistic (Brooks & Gelman, 1998), which was below 1.05 for all parameters.^9^ We obtained ten independent sets of posterior samples for both the full and the restricted model, which were used to assess the uncertainty of the Bayes factor estimates.

Once the posterior samples were obtained, we computed the Bayes factor in favor of the full model using the Warp-III, the simple Monte Carlo, the Savage–Dickey, and the RJMCMC methods. The implementations of the four approaches are available at https://osf.io/ynwpa/. To assess the uncertainty of the Bayes factor estimates, we repeated each procedure ten times for each model. For the Warp-III, Savage–Dickey, and RJMCMC methods, we used a fresh set of posterior samples for each repetition.

### Results

**Fig. 3:**
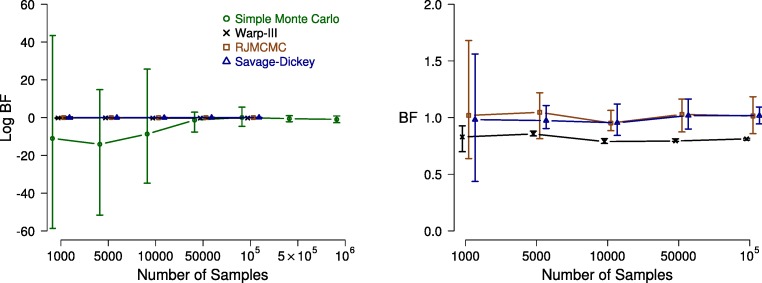
Bayes factor estimates for the single-participant case as a function of the number of samples. The *left panel* displays the *log* Bayes factor estimates computed using the Warp-III (*black crosses*), simple Monte Carlo (*green circles*), Savage–Dickey (*blue triangles*), and RJMCMC (*brown squares*) methods. The *right panel* displays the Bayes factor estimates computed using the Warp-III (*black crosses*), Savage–Dickey (*blue triangles*), and RJMCMC (*brown squares*) methods (i.e., omitting the simple Monte Carlo estimates and displaying the results on the Bayes factor and not log Bayes factor scale). For Warp-III, the *x*-axis corresponds to the number of posterior samples (collapsed across all chains) used for computing the marginal likelihood for each model. For simple Monte Carlo, it corresponds to the number of prior samples used for computing the marginal likelihoods. For Savage–Dickey, it corresponds to the number of posterior samples used to estimate the density of the posterior distribution at the test value (i.e., 3.55). For RJMCMC, it corresponds to the number of posterior samples used from each model (for details, see the Appendix). The *symbols* (i.e., crosses, circles, triangles, squares) indicate the median (log) Bayes factor estimates and *bars* indicate the range of the estimates across the ten repetitions. Available at https://tinyurl.com/y5brs44a under CC license https://creativecommons.org/licenses/by/2.0/

The left panel of Fig. 3 displays estimates of the log Bayes factor as a function of the number of samples. Note that we included an order of magnitude more samples for the simple Monte Carlo method in order to produce results that are comparable to estimates provided by the other methods. The right panel of Fig. 3 zooms in on the results obtained with the Warp-III, Savage–Dickey, and RJMCMC methods and omits the simple Monte Carlo estimates; this panel shows the Bayes factor and *not* the log Bayes factor to facilitate interpretation.

All four methods eventually converged to a log Bayes factor estimate close to zero (equivalently, a Bayes factor estimate close to one). As the number of samples increased, the uncertainty of the estimates decreased. For this example, Warp-III resulted in the smallest uncertainty intervals. The Warp-III, Savage–Dickey, and RJMCMC methods resulted in stable Bayes factor estimates already with 1000 samples. Although the three methods numerically did not yield the exact same Bayes factors, they all produced estimates close to one with relatively small uncertainty. The simple Monte Carlo method was clearly the least efficient; it produced wide uncertainty intervals and took approximately 50,000-100,000 samples to converge to the estimates from the other methods. Note that the number of samples required by the different methods for the stable and reliable estimation of the Bayes factor may vary depending on the characteristics of the specific example and should not be interpreted as a guideline.

Although in this particular example we were able to obtain stable and accurate Bayes factor estimates with all four methods, this is not necessarily the case for more complicated—non-nested and hierarchical—model selection problems. The Savage–Dickey method cannot be used for non-nested model comparison. Moreover, the Savage–Dickey estimate of the Bayes factor becomes very unstable if the test value falls in the tail of the posterior distribution because density estimates in the tails of the posterior are highly variable. Similarly, the RJMCMC approach cannot be easily generalized to situations involving non-nested comparisons. RJMCMC exploits the relations between the parameters of the models; however, if the models are non-nested, it might be impossible to relate the two sets of parameters. Even generalizing RJMCMC to nested hierarchical comparisons is challenging because it involves linking a large number of parameters, especially if the vector of participant-level parameters differs between the two models for each participant. Furthermore, as a result of the strong parameter correlations in evidence-accumulation models, fixing one parameter in nested model comparisons can lead to substantial changes in the other parameters, making it even more difficult to efficiently link the competing models. Because of these challenges associated with non-nested and hierarchical model comparisons, we believe that the Savage–Dickey density ratio and RJMCMC methods are not suited as general model selection tools for evidence-accumulation models and will not be considered further.

The simple Monte Carlo and the Warp-III method can be used for both nested and non-nested model comparisons because they consider one model at a time.^10^ In Warp-III, this also allows us to use a convenient proposal distribution chosen to maximize the overlap between the proposal and the posterior, which leads to a substantial gain in efficiency relative to simple Monte Carlo sampling. The inefficiency of simple Monte Carlo in our straightforward single-participant example suggests that this method is infeasible in many practical applications of hierarchical evidence-accumulation models. First, as also acknowledged by Evans and Brown (2018), simple Monte Carlo can result in highly variable Bayes factor estimates in hierarchical settings. Second, the number of samples needed to obtain stable estimates with simple Monte Carlo sampling can quickly become unmanageable. This was indeed the case when we tried to apply it to the hierarchical model comparison problems outlined in the next section.^11^

## Simulation study II: nested and non-nested model comparison for the hierarchical case

As a second example, we considered eight LBA data sets that featured observations from multiple participants generated and fit using the hierarchical approach. We investigated the performance of Warp-III for two nested and two non-nested model comparison problems.

### Models and data

We simulated a design with four cells, two conditions that differed in a particular parameter crossed with two stimuli, and two possible responses. In the nested case, we compared a model that allowed only mean drift rate *v*_true_ to be different across conditions (i.e., *V* -model) to a null model that featured one common *v*_true_ parameter for both conditions (i.e., 0-model). In the non-nested case, we compared the *V* -model to a model that allowed only threshold *B* to be different across conditions (i.e., *B*-model). Note that we made these comparisons in both directions, for example, we computed the Bayes factor for the *V* -model vs. *B*-model comparison when the *V* -model generated the data, and computed the Bayes factor for the *B*-model vs. *V* -model comparison when the *B*-model generated the data.

We generated new data sets from both models in each comparison. We used two different combinations of the number of participants (*n*) and the number of trials per cell (*k*), both with 4000 data points in total. Thus, overall, there were eight different data sets: one for each of the four comparisons at each group size. In the first combination, we simulated data using *n* = 20 with *k* = 200, corresponding to a smaller group of participants each measured fairly well. In the second combination, we simulated data using *n* = 80 with *k* = 50, corresponding to a larger group of participants each measured at or below the lower bound of *k* required for acceptable individual estimation. These two cases exemplified either an emphasis on individual or group estimation. In the former case, the number of participants was at the lower bound of *n* required for acceptable estimation of the group-level parameters. In the latter case, estimation of the participant-specific parameters relied heavily on the additional constraint provided by the hierarchical structure.

To generate the data sets, we used normal group-level distributions for each parameter (truncated below to allow only positive values), specified the location (*μ*) and scale (*σ*) of the group-level distributions, and then simulated participant-specific parameters from these normal distributions. Subsequently, the participant-specific parameters were used to generate trials for each participant. To ensure identifiability, the standard deviation of the drift rate corresponding to the accumulator for the correct response, *s*_true_, was fixed to one for every participant.

To generate data from the *V* -model, we used the following *μ* parameters (where bracketed superscripts indicate experimental condition): $\mu _{A} = 1$, *μ*_*B*_ = 0.4, $\mu _{v_{\text {true}}^{(1)}} = 4$, $\mu _{v_{\text {true}}^{(2)}} = 3$, $\mu _{v_{\text {false}}} = 1$, $\mu _{s_{\text {false}}} = 1$, and $\mu _{t_{0}} = 0.3$. For the 0-model, we used *μ*_*A*_ = 1, *μ*_*B*_ = 0.4, $\mu _{v_{\text {true}}} = 3$, $\mu _{v_{\text {false}}} = 1$, $\mu _{s_{\text {false}}} = 1$, and $\mu _{t_{0}} = 0.3$. For the *B*-model, we used $\mu _{A} = 1$, $\mu _{B^{(1)}} = 0.3$, $\mu _{B^{(2)}} = 0.7$, $\mu _{v_{\text {true}}} = 3.5$, $\mu _{v_{\text {false}}} = 1$, $\mu _{s_{\text {false}}} = 1$, and $\mu _{t_{0}} = 0.3$. The data-generating *σ* parameters were obtained by dividing the *μ* parameters by ten, resulting in appreciable but not excessive individual differences in the participant-specific parameters.

### Prior distributions

We used zero-bounded truncated normal group-level distributions to model individual differences in the parameters. We used the following prior distributions for the group-level parameters:

9 $$ \begin{array}{@{}rcl@{}} \mu_{A}, \sigma_{A} &\sim& \mathcal{N}_{+}(1, 1)\\ \mu_{B}, \sigma_{B} &\sim& \mathcal{N}_{+}(0.4, 0.4^{2}) \\ \mu_{v_{\text{true}}}, \sigma_{v_{\text{true}}} &\sim& \mathcal{N}_{+}(3, 3^{2}) \\ \mu_{v_{\text{false}}}, \sigma_{v_{\text{false}}} &\sim& \mathcal{N}_{+}(1, 1) \\ \mu_{s_{\text{false}}}, \sigma_{s_{\text{false}}} &\sim& \mathcal{N}_{+}(1, 1) \\ \mu_{t_{0}}, \sigma_{t_{0}} &\sim &\mathcal{N}_{+}(0.3, 0.3^{2}). \end{array} $$

As for the single-participant case, we believe that the priors provide a reasonable setup but they may be replaced by empirically informed priors in future applications.

### Parameter estimation and model comparison

We used the DE-MCMC algorithm, as implemented in the DMC software to estimate the model parameters. We first estimated parameters separately for each synthetic participant, similar to our previous single-participant example. The result of this phase provided the starting values for the hierarchical analysis. For each model, we set the number of MCMC chains to three times the number of participant-specific parameters. We thinned each MCMC chain to retain only every 10th posterior sample. Burn-in was accomplished by DMC’s *h.run.unstuck.dmc* function with a 5% migration probability. We then used the *h.run.converge.dmc* function with no migration until 250 iterations were obtained that appeared to be converged to the stationary distribution ($\hat {R} < 1.1$). Further iterations were then added using the *h.run.dmc* function until we obtained approximately 100,000 posterior samples per parameter (the exact number of samples varied because the number of MCMC chains varied among the different models). With this very large number of samples, $\hat {R}$ was very close to 1 for all parameters at both the group and participant levels. We obtained ten independent sets of posterior samples for each model, which were used to assess the uncertainty of the Bayes factor estimates.

Once the posterior samples were obtained, we computed the Bayes factor in favor of the data-generating models using Warp-III.^12^ For each model, we assessed the uncertainty of the estimates by running the Warp-III sampler ten times using a fresh set of posterior samples for each repetition.

### Results

**Fig. 4:**
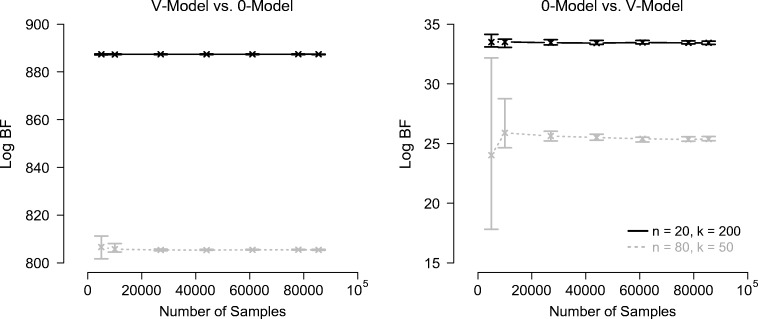
Log Bayes factor estimates obtained with Warp-III sampling for the nested hierarchical model comparisons as a function of the number of posterior samples (collapsed across all chains) used for computing the marginal likelihood for each model. *Crosses* indicate the median log Bayes factor estimates and *bars* indicate the range of the estimates across the ten repetitions. The *left panel* shows results for the data sets generated from the *V* -model; the *right panel* shows results for the data sets generated from the 0-model. Results for *n* = 20 with *k* = 200 are displayed in *black*; results for *n* = 80 with *k* = 50 are displayed *in gray with dotted lines*. The log Bayes factor is expressed in favor of the data-generating model. Available at https://tinyurl.com/yxgsgjaw under CC license https://creativecommons.org/licenses/by/2.0/

**Fig. 5:**
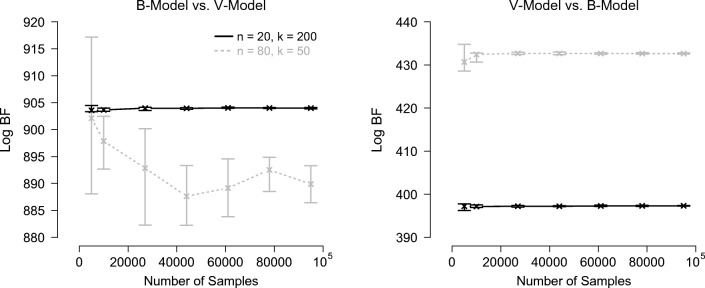
Log Bayes factor estimates obtained with Warp-III sampling for the non-nested hierarchical model comparisons as a function of the number of posterior samples (collapsed across all chains) used for computing the marginal likelihood for each model. *Crosses* indicate the median log Bayes factor estimates and *bars* indicate the range of the estimates across the ten repetitions. The *left panel* shows results for the data sets generated from the *B*-model; the *right panel* shows results for the data sets generated from the *V* -model. Results for *n* = 20 with *k* = 200 are displayed in *black*; results for *n* = 80 with *k* = 50 are displayed *in gray with dotted lines*. The log Bayes factor is expressed in favor of the data-generating model. Available at https://tinyurl.com/y3f7l263https://tinyurl.com/y3f7l263 under CC license https://creativecommons.org/licenses/by/2.0/

Figure 4 shows the log Bayes factor estimates obtained with Warp-III sampling as a function of the number of samples for the nested comparisons and Fig. 5 shows the results for the non-nested comparisons.^13^ The log Bayes factors are expressed in favor of the data-generating models.

The figures illustrate that Warp-III resulted in stable Bayes factor estimates in favor of the data-generating model with narrow uncertainty intervals in all but one case, the non-nested *B*-model vs. *V* -model comparison for the *n* = 80 with *k* = 50 data set. For this data set, the iterative scheme from Eq. 5 initially did not seem to converge, but instead oscillated between two different values, say *x*_1_ and *x*_2_. We were able to achieve convergence by stopping the iterative scheme and re-starting it with the initial guess of the marginal likelihood set to the geometric mean of the two values between which the estimate initially oscillated (i.e., the square root of the product of *x*_1_ and *x*_2_). Although this approach enabled us to obtain an estimate of the marginal likelihood, the uncertainty of this estimate was noticeably larger than for the other cases. Nevertheless, this estimate was sufficiently certain to conclude that the Bayes factor clearly favored the *B*-model.^14^

The results show that the hierarchical model comparisons required substantially more samples than the single-participant case. Note also that more samples were needed for the *n* = 80 with *k* = 50 data sets than for the *n* = 20 with *k* = 200 data sets to obtain comparable uncertainty intervals. The reason is that the number of participants, *n*, determines how many participant-specific parameters need to be integrated out, whereas the number of trials per cell, *k*, does not affect the number of model parameters. Therefore, increasing the number of participants increases the dimensionality of the integral in Eq. 2 that is estimated via Warp-III. It is likely that the greater difficulty in obtaining well-behaved participant-specific parameter estimates with *k* = 50 has also contributed to the larger uncertainty intervals.

All Bayes factors yielded overwhelming evidence for the data-generating model, including the ones computed for the data sets generated from the nested 0-model (i.e., right panel of Fig. 4). Note, however, that the magnitude of the Bayes factors for these nested examples is smaller than for the other examples. This result is not unexpected: the *V* -model can account for all data sets that the 0-model can account for and, additionally, also for data sets that show a difference in *v*_true_ between conditions. Therefore, the Bayes factor can only favor the 0-model due to parsimony and not because it describes the data better than the *V* -model. Note also that although the Bayes factors clearly favored the data-generating models, this may not necessarily be the case in other examples. As outlined in our earlier discussion of model inversion, Bayesian inference conditions on the data at hand and it may be reasonable to obtain evidence in favor of a different model than the data-generating one for certain data sets.

## Simulation study III: estimating equivocal Bayes factors for the hierarchical case

In the previous section, it was demonstrated that Warp-III yields stable and precise Bayes factor estimates for different hierarchical examples. Many of these Bayes factor estimates were very large and it could be argued that for large Bayes factors, obtaining very precise estimates is not crucial since the qualitative conclusion (“overwhelming evidence”) will not change unless the estimation uncertainty is extremely large. In this section, we demonstrate that Warp-III is also able to provide precise estimates of a Bayes factor close to 1 for the hierarchical case. Estimating Bayes factors in this range precisely is important since a large estimation uncertainty would make it difficult to judge which model is favored.

### Models and data

For this example, we reused the data set generated from the *B*-model with *n* = 20 and *k* = 200 described in the previous section. We compared the data-generating *B*-model to a restricted *B*_res_-model. The *B*_res_-model was identical to the *B*-model except that the group-level parameter $\mu _{v_{\text {false}}}$ was fixed to 1.24. This value was chosen to yield a Bayes factor close to 1.^15^

### Prior distributions

The prior distributions were identical to the ones used in the previous hierarchical example. Note that for the *B*_res_-model, the group-level parameter $\mu _{v_{\text {false}}}$ was fixed to 1.24 and was not assigned a prior distribution.

### Parameter estimation and model comparison

Parameter estimation and model comparison was conducted in an analogous manner to the previous hierarchical example. Note that we reused the log marginal likelihood estimates for the *B*-model from the previous example which was based on the exact same data set.

### Results

**Fig. 6:**
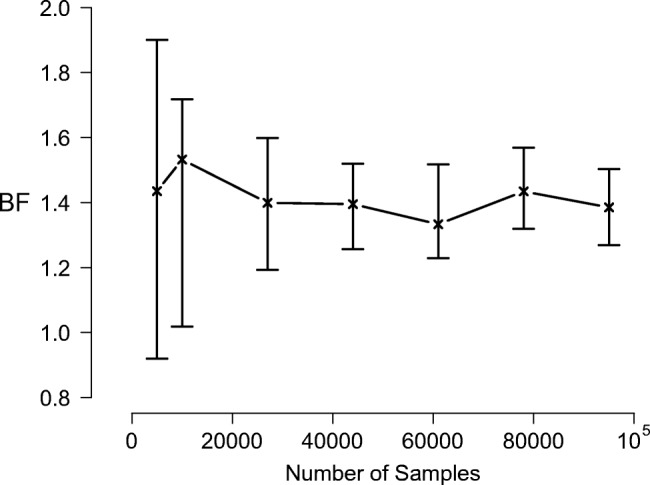
Bayes factor estimates obtained with Warp-III sampling for the *B*-model vs. *B*_res_-model example as a function of the number of posterior samples (collapsed across all chains) used for computing the marginal likelihood for each model. *Crosses* indicate the median Bayes factor estimates and *bars* indicate the range of the estimates across the ten repetitions. The data set was generated from the *B*-model with *n* = 20 and *k* = 200 and is identical to the one used in the left panel of Fig. 5. The Bayes factor is expressed in favor of the data-generating model. Available at https://tinyurl.com/y599st45 under CC license https://creativecommons.org/licenses/by/2.0/

Figure 6 shows the Bayes factor (*not* log Bayes factor) estimates obtained with Warp-III sampling as a function of the number of samples. The Bayes factor is expressed in favor of the data-generating *B*-model. The figure illustrates that Warp-III resulted in stable Bayes factor estimates with narrow uncertainty intervals. The estimated Bayes factor is slightly larger than 1 indicating that the data-generating *B*-model is slightly favored. Nevertheless, a Bayes factor close to 1 indicates that none of the models is favored in a compelling fashion by the data at hand; the evidence is ambiguous.

## Discussion

Over the last decade, the Bayesian estimation of evidence-accumulation models has gained momentum (e.g., Heathcote et al.,, 2018; Vandekerckhove et al.,, 2011; Wiecki et al.,, 2013). This increase in popularity is largely attributable to the advantages afforded by the Bayesian hierarchical framework that allows researchers to obtain well-constrained parameter estimates even in situations with relatively few observations per participant. Despite recent advances in the Bayesian estimation of evidence-accumulation models, model comparison continues to rely on suboptimal procedures, such as posterior parameter inference and model selection criteria known to favor overly complex models.

In this paper, therefore, we advocated model selection for evidence-accumulation models based on the Bayes factor (e.g., Etz & Wagenmakers, 2017; Kass & Raftery, 1995; Ly et al.,, 2016; Jeffreys, 1961). The Bayes factor is given by the ratio of the marginal likelihoods of the competing models and thus enables the quantification of relative evidence on a continuous scale (e.g., Wagenmakers et al.,, 2018). The Bayes factor implements a trade-off between parsimony and goodness-of-fit (Jefferys & Berger, 1992; Myung & Pitt, 1997) and is considered as “the standard Bayesian solution to the hypothesis testing and model selection problems” (Lewis & Raftery, 1997, p. 648). Bayes factors enable the computation of posterior model probabilities, which provide an intuitive metric for comparison among models. Bayes factors also enable Bayesian model averaging, which avoids the need to make categorical decisions between models and which produces better calibrated predictions (e.g., Hoeting, Madigan, Raftery, & Volinsky, 1999). Bayes factors are well suited for the type of model comparison problems that are faced by cognitive modelers because they do not favor overly complex models, and so guard against the proliferation of “crud factors” that plague psychology (Meehl, 1990).

Despite the advantages afforded by the Bayesian framework, Bayes factors are rarely, if ever, used for evidence-accumulation models, largely because of the computational challenges involved in the evaluation of the marginal likelihood. Here we advocated Warp-III bridge sampling (Meng & Schilling, 2002) for computing the marginal likelihood—and hence the Bayes factor—for evidence-accumulation models. We believe that Warp-III is well suited for cognitive models in general and evidence-accumulation models in particular because, as we have shown, it can be straightforwardly applied to hierarchical models and non-nested comparisons, unlike the simple Monte Carlo and the Savage–Dickey approaches. Moreover, Warp-III is relatively easy to implement, and requires only the posterior samples routinely collected during parameter estimation. In contrast to transdimensional MCMC methods, such as RJMCMC, it does not require changing the sampling algorithm or linking the competing models, which can be problematic for hierarchical and non-nested models. We have shown that Warp-III bridge sampling is practically feasible even in complex and high-dimensional hierarchical instantiations of the Linear Ballistic Accumulator (LBA; Brown & Heathcote, 2008). Although we encountered a challenging case with scarce participant-level data (left panel of Fig. 5), even in that case we were able to detect and ameliorate the convergence problem.

Once the posterior samples are obtained, computing the marginal likelihood for the single-participant case using Warp-III is relatively fast. For each repetition, it took approximately 13 min to run the Warp-III sampler with 100,000 posterior samples, using four CPU cores on our servers. As these servers are old and the individual cores relatively slow given they are embedded in 16-core chips, more modern quad-core laptops will achieve the task in a much shorter time. Naturally, in the hierarchical setting, the computational burden is higher and strongly depends on the number of participants. For instance, for the *V* -model vs. *B*-model comparison (right panel in Fig. 5) in combination with *n* = 20 and *k* = 200, running the Warp-III sampler with 95,000 posterior samples took approximately 7 hours, using four CPU cores on our servers. In contrast, for the *n* = 80 and *k* = 50 case, the computational time was approximately 25 hours. However, it is important to note that it was not necessary to collect such a high number of posterior samples. For the individual case, the Bayes factor estimate was precise and stable after only 1000 samples. For most hierarchical comparisons, we obtained well-behaved Bayes factor estimates with approximately 20,000–30,000 samples. Note also that the computational time strongly depends on the specific programming language used for evaluating the likelihood and the prior. Our implementation relies on R (R Core Team, 2019), but integrating the Warp-III sampler with Lin & Heathcote’s (2017) C++ implementation of the LBA and the DDM is expected to speed up sampling by an order of magnitude. In summary, although Warp-III is computationally more intensive than using model selection criteria such as the DIC (Spiegelhalter et al., 2002), in standard applications of evidence-accumulation models, the computational costs are manageable, even using personal computers. We believe that the computational costs of Warp-III are a small price to pay for the advantages afforded by the use of principled Bayesian model selection techniques. Where practical issues are faced due to the need to select among a large number of models, researchers may consider an initial triage using easy-to-compute alternatives, such as DIC, in order to obtain a candidate set for model selection based on Bayes factors (for related approaches, see Madigan & Raftery, 1994, and Overstall & Forster, 2010).

As many evidence-accumulation models have analytic likelihoods, and so are amenable to MCMC methods for obtaining posterior distributions, Warp-III sampling is not limited to the LBA, but may be readily applied to other models, such as the diffusion decision model (DDM; Ratcliff, 1978; Ratcliff & McKoon, 2008). Heathcote et al.,’s (2018) DMC software enables the hierarchical MCMC-based estimation of not only the LBA and the DDM, but also a variety of other models including single-boundary and racing diffusion models (Leite & Ratcliff, 2010; Tilman et al.,, 2017; Logan, Van Zandt, Verbruggen, & Wagenmakers, 2014), lognormal race models (Heathcote & Love, 2012; Rouder, Province, Morey, Gómez, & Heathcote, 2015), as well as race models of the stop-signal paradigm (Matzke et al.,, 2013; Matzke, Love, & Heathcote, 2017). Our easy-to-use R-implementation of the Warp-III sampler enables the computation of the marginal likelihood of any model implemented in the DMC software. When analytic likelihoods are not available, approximate Bayesian computation may be used to enable MCMC sampling, opening up the possibility to explore more complex and realistic cognitive process models (Turner & Sederberg, 2014; Holmes, Trueblood, & Heathcote, 2016), although this approach remains challenging (e.g., Lin & Heathcote, 2018). Future research should investigate the performance of simulation-based methods, such as Warp-III, in the context of models without analytic likelihood.

As illustrated in our single-participant example, the Bayes factor will not necessarily select a data-generating model. In contrast, as explained in detail before, it might be the case that the Bayes factor favors a model different than the data-generating one for certain data sets. However, in the single-participant example and in the final hierarchical example, the Bayes factor did not clearly favor a model different than the data-generating one but was approximately 1, meaning that both models were about equally likely. Thus, another advantage of Bayes factors is that they allow one to disentangle evidence of absence (i.e., the Bayes factor favors the simpler model) and absence of evidence (i.e., the Bayes factor is approximately 1).

It is crucial to acknowledge that the Bayes factor critically depends on the prior distribution of the model parameters. We emphasize that the priors we used in the present article are not the gold standard for the LBA. We are presently developing empirically informed prior distributions for the LBA and the DDM based on archival data sets. In the meantime, we recommend that researchers develop their own empirically based priors (perhaps through pilot work or analysis of related archival data sets) in LBA applications. For the DDM, the distributions of parameter values in Matzke and Wagenmakers (2009) already provide reasonable priors. We see the development of theoretically and empirically informed prior distributions as necessary part of the maturation of any well-specified quantitative model, consistent with the position of Lee and Vanpaemel (2018).

### Practical recommendations

In this final section, we provide recommendations about the use of Warp-III sampling in practical applications. Our recommendations should not be interpreted as strict guidelines, but rather as suggestions based on our experience of using Warp-III in the context of cognitive models in general and evidence-accumulation models in particular.

#### How to assess the uncertainty and stability of the estimate

Once the data have been observed and the model (i.e., the likelihood and the prior) have been specified, there is a single *true* marginal likelihood corresponding to a particular data-model combination. However, for (hierarchical) evidence-accumulation models, the true marginal likelihood cannot be computed analytically and must be estimated. As with all estimates, the marginal likelihood provided by Warp-III is uncertain and may vary even for the same data-model combination. Consequently, it is crucial to assess and report the uncertainty of the estimate and investigate the degree to which uncertainty affects conclusions.

Our recommendation is to assess the uncertainty directly for the quantity of interest. For instance, when conclusions are based on the Bayes factor, researchers should assess the uncertainty of the Bayes factor; when conclusions are based on posterior model probabilities, researchers should assess the uncertainty of the posterior model probabilities. To do so, we recommend researchers to compute the quantity of interest repeatedly based on independent runs of Warp-III. For example, when one is interested in estimating the Bayes factor, one should repeatedly (1) draw fresh posterior samples from the competing models; (2) use Warp-III to estimate the marginal likelihood of the models; and (3) compute the resulting Bayes factor. The uncertainty of the estimate can then be assessed by considering the empirical variability of the Bayes factor estimates across the repetitions. The empirical assessment of uncertainty is generally considered as the gold standard, even when approximate errors are available such as for the simple multivariate normal bridge sampling estimator (e.g., Frühwirth–Schnatter, 2006).^16^

We find it useful to not only assess the uncertainty, but also to investigate whether the estimate of the quantity of interest (e.g., Bayes factor) has stabilized. As our simulations demonstrated, when successively increasing the number of samples, the estimate becomes more precise and—after some initial fluctuation—tends to stabilize. One way to assess stability is to compute the quantity of interest using batches of the available posterior samples, as we have done in our simulations. However, we acknowledge that this process can be time consuming. A crude alternative is to compute the estimate with the corresponding uncertainty based on (at least) three different samples sizes, for instance, (a) $\frac {1}{3}$, (b) $\frac {2}{3}$, and (c) all of the posterior samples. Considering the sequence of these three estimates allows one to get an idea about whether the estimate has stabilized.

#### How many samples are required for precise and stable estimates

Assessing the uncertainty and stability of the estimate is a natural and—in our opinion—the best approach to determine the number of samples required for reliable conclusions. Note that the required level of precision and stability depends on the particular application. For instance, for one of our non-nested hierarchical examples (left panel in Fig. 5), the Bayes factor estimates were relatively uncertain and fluctuated quite substantially even in the high-sample region. However, given that all of the estimates provided overwhelming evidence for the *B*-model, the achieved accuracy and stability were sufficiently high to conclude that the *B*-model was clearly favored over the *V* -model. In contrast, in situations when the Bayes factor estimates do not provide compelling evidence for either model (for instance, when the Bayes factor estimates are varying around 1), it is crucial to obtain more precise and stable estimates to ensure that fluctuations do not influence which of the two models is favored or whether it is concluded that the evidence is equivocal. The single-participant and the final hierarchical example indicate that it is possible to obtain precise and stable Warp-III Bayes factor estimates also for this Bayes factor range.

Given these considerations, combined with the fact that the quality of the estimate depends on factors such as the number of participants and the complexity of the models, we are unable to provide general recommendations about the number of samples necessary for the reliable application of Warp-III sampling. Warp-III requires more posterior samples than one would typically collect for the purpose of parameter estimation. In our experience, a minimum of 1000-2000 posterior samples (collapsed across chains) typically provides a reasonable starting point in single-participant applications. In hierarchical applications, we recommend at least 10,000–20,000 samples. Nevertheless, as with all simulation-based methods, the more samples, the better. Note that our recommendations assume that the posterior samples are not highly auto-correlated; the degree of thinning in our simulations resulted in posterior samples that were virtually uncorrelated. Although autocorrelation is not itself necessarily a problem for parameter estimation, it does reduce the effective number of samples, and when large numbers of samples are required it is practically efficient to thin the samples, at least to the degree that there is little loss of effective sample size. Warp-III also benefits from having posterior samples with low autocorrelation. One reason is that the “optimal” bridge function is only optimal in case the posterior samples are independent and identically distributed which is not the case when using MCMC methods. However, some autocorrelation may not be too worrisome since, in our implementation, we use an effective sample size in this bridge function.

#### When to use simple bridge sampling and when to use Warp-III sampling

The Warp-III estimator is an advanced version of the “simple” multivariate normal bridge sampling estimator (e.g., Overstall & Forster, 2010). Warp-III matches the first three moments of the posterior and the proposal distribution; the multivariate normal approach—which is equivalent to Warp-II—matches only the first two moments of the distributions. As the precision of the estimate of the marginal likelihood is governed by the overlap between the posterior and the proposal distribution, the Warp-III estimate is at least as precise as the estimate computed using simple bridge sampling.^17^ With symmetric posterior distributions, the advantage of Warp-III diminishes, but nothing is lost in terms of precision relative to simple bridge sampling. In contrast, with skewed posterior distributions, Warp-III results in more precise estimates because it is able to match the posterior and the proposal more closely. Note that both Warp-III and simple bridge sampling assume that the posterior samples are allowed to range across the entire real line. Hence, the skew of the posterior distributions must be assessed after the appropriate transformations. This does not mean that sampling from the posterior distributions must occur with all parameters transformed to the real line. In fact, in our simulations, only the *v* parameters were sampled on the real line; all other parameters were transformed to the real line after the posterior samples have been obtained. Our R-implementation of the Warp-III sampler automatically applies the appropriate transformations to the posterior samples obtained with the DMC software. Specifically, the implementation assumes that each posterior component can be transformed separately^18^ and distinguishes between four different parameter types: (1) unbounded parameters, (2) lower-bounded parameters, (3) upper-bounded parameters, and (4) double-bounded parameters (i.e., parameters that have a lower and an upper bound). Table 1 displays the transformations that are used for the different parameter types. After having detected the parameter type, an appropriate transformation is applied and the expressions are adjusted by the relevant Jacobian contribution (see Table 1).

**Table 1:** Overview of the transformations used in the Warp-III implementation. *𝜃*_*i*_ denotes a parameter and *ω*_*i*_ denotes the corresponding new parameter that is obtained after having transformed *𝜃*_*i*_ to the real line. *l* denotes a parameter lower bound and *u* denotes an upper bound. Φ(⋅) denotes the cumulative distribution function and *ϕ*(⋅) the probability density function of the normal distribution. The table displays the parameter type, the corresponding transformation, inverse-transformation, and the relevant Jacobian contribution

Type	Transformation	Inv.-Transformation	Jacobian contribution
unbounded	*ω*_*i*_ = *𝜃*_*i*_	*𝜃*_*i*_ = *ω*_*i*_	$\left \lvert \frac {\partial \theta _{i}}{\partial \omega _{i}}\right \rvert = 1$
lower-bounded	$\omega _{i} = \log \left (\theta _{i} - l\right )$	$\theta _{i} = \exp \left (\omega _{i}\right ) + l$	$\left \lvert \frac {\partial \theta _{i}}{\partial \omega _{i}}\right \rvert = \exp \left (\omega _{i}\right )$
upper-bounded	$\omega _{i} = \log \left (u - \theta _{i}\right )$	$\theta _{i} = u - \exp \left (\omega _{i}\right )$	$\left \lvert \frac {\partial \theta _{i}}{\partial \omega _{i}}\right \rvert = \exp \left (\omega _{i}\right )$
double-bounded	$\omega _{i} = {\Phi }^{-1}\left (\frac {\theta _{i} - l}{u - l}\right )$	$\theta _{i} = \left (u - l\right ){\Phi }\left (\omega _{i}\right ) + l$	$\left \lvert \frac {\partial \theta _{i}}{\partial \omega _{i}}\right \rvert = \left (u - l\right ) \phi \left (\omega _{i}\right )$

In general, Warp-III is a more powerful tool than simple bridge sampling for estimating the marginal likelihood, but the gain in precision depends on the particular application. A potential advantage of simple bridge sampling is its relative speed. Warp-III results in a mixture representation which requires one to evaluate the un-normalized posterior twice as often as in simple bridge sampling (e.g., Gronau et al.,, 2019; Overstall, 2010). This implies a speed–accuracy trade-off: simple bridge sampling may be less precise but faster; Warp-III may be more precise but slower. Of course, one may increase the precision of the simple bridge sampling estimate by increasing the number of posterior samples. However, this approach neglects the fact that—in evidence-accumulator models in particular—obtaining the posterior samples typically takes substantially longer than computing the marginal likelihood using Warp-III. Therefore, although simple bridge sampling is faster for a given (initial) set of posterior samples, it is not necessarily true that it is more efficient to run the simpler version based on additional posterior samples than to run Warp-III on the initial set of samples to obtain comparable precision. Furthermore, we expect that the problem of seemingly non-converging estimates may be more frequent when using simple bridge sampling. Although this can be addressed by restarting the iterative scheme from an appropriately chosen start value, as shown in the left panel of Fig. 5, this solution substantially increases the uncertainty of the estimate.

In situations where the joint posterior is exactly multivariate normal,^19^ simple bridge sampling is clearly more efficient than Warp-III. However, it is challenging to assess multivariate normality in the high-dimensional spaces regularly encountered in hierarchical evidence-accumulation models. Although evaluating the marginal posterior distributions is feasible in most standard applications, normality of the marginals—which is often not the case for evidence-accumulation models applied to scarce data—does not necessarily imply that the joint posterior is multivariate normal. In sum, if one expects multivariate normal posterior distributions, simple bridge sampling is more efficient and should be preferred. Whenever this is not the case, we recommend Warp-III sampling.

## Conclusions

In this article, we advocated Warp-III bridge sampling as a general method for estimating the marginal likelihood—and hence the Bayes factor—for evidence-accumulation models. We demonstrated that Warp-III sampling provides a powerful and flexible approach that can be applied to both nested and non-nested model comparisons and—once posterior samples from the competing models have been obtained—it is straightforward to implement even in hierarchical settings. We believe that our easy-to-use and freely available implementation of Warp-III sampling will greatly facilitate the use of principled Bayesian model selection in practical applications of evidence-accumulation models.

## Open Practice Statement

R scripts for reproducing the results presented in this manuscript are available at https://osf.io/ynwpa/.

## Appendix A: Savage–Dickey density ratio

Suppose that the parameter vector ***𝜃*** can be partitioned into a set of nuisance parameters ***ζ*** and test-relevant parameters ***η*** so that ***𝜃*** = (***ζ***,***η***). The Savage–Dickey density ratio (Dickey & Lientz, 1970; Wagenmakers et al., 2010) can then be used to compute the Bayes factor for testing whether ***η*** is equal to a constant ***η***_0_ in the presence of nuisance parameters ***ζ***. Concretely, the Bayes factor compares model ${\mathscr{M}}_{0}$, which assigns ***ζ*** the prior density *p*_0_(***ζ***) and fixes ***η*** to the constant ***η***_0_ to model ${\mathscr{M}}_{1}$ which assigns ***ζ*** and ***η*** the joint prior density *p*_1_(***ζ***,***η***). The Savage–Dickey density ratio representation of the Bayes factor is then given by

10 $$ \text{BF}_{01} = \frac{p_{1}(\boldsymbol{\eta}_{0} \mid \boldsymbol{y})}{p_{1}(\boldsymbol{\eta}_{0})}, $$

where *p*_1_(***η***_0_∣***y***) denotes the marginal posterior density of ***η*** under ${\mathscr{M}}_{1}$ evaluated at ***η***_0_ and *p*_1_(***η***_0_) denotes the marginal prior density of ***η*** under ${\mathscr{M}}_{1}$ evaluated at ***η***_0_. Note that this representation is only valid in case *p*_1_(***ζ***∣***η***_0_) = *p*_0_(***ζ***). Hence, conditional on ***η*** = ***η***_0_, the prior density for ***ζ*** under ${\mathscr{M}}_{1}$ must be identical to the prior density of ***ζ*** under ${\mathscr{M}}_{0}$.^20^ In our single-participant example, this assumption holds since the prior under ${\mathscr{M}}_{1}$ is given by *p*_1_(***ζ***,***η***) = *p*_0_(***ζ***)*p*_1_(***η***). We used a logspline density estimator (Kooperberg, 2016) to estimate the marginal posterior density at the point of interest.

## Appendix B: Reversible jump Markov chain Monte Carlo

Reversible jump Markov chain Monte Carlo (RJMCMC; Green, 1995) refers to an MCMC sampler on an enlarged state space, which incorporates a model indicator *M* as an additional unknown. The posterior of the model indicator *M* can be used to estimate posterior model probabilities and posterior model odds. An estimate of the Bayes factor can be obtained by dividing the estimated posterior model odds by the known prior model odds. Barker and Link (2013) described a version of RJMCMC that represents the process intuitively as a Gibbs sampler where updates of the model indicator *M* are alternated with updates of a “palette” parameter vector ***ψ***. The palette vector ***ψ*** has dimension $d = {\max \limits } \left \{\dim (\boldsymbol {\theta }_{k})\right \}$ where ***𝜃***_*k*_ denotes the parameter vector for model *M*_*k*_, *k* = 1, 2,…,*K* and *K* denotes the number of models under consideration.^21^ Each model’s parameter vector ***𝜃***_*k*_ can be obtained from the palette vector ***ψ*** by a known invertible mapping *g*_*k*_(***ψ***) = ***ξ***_*k*_ = (***𝜃***_*k*_,***u***_*k*_), where ***u***_*k*_ denotes a vector of auxiliary variables which is redundant to model *M*_*k*_ but ensures that the dimensionality of ***ψ*** and ***ξ***_*k*_ matches.

The full-conditional distributions for the Gibbs sampler are determined by the joint model *p*(***y***,***ψ***,*M*) = *p*(***y***∣***ψ***,*M*)*p*(***ψ***∣*M*)*p*(*M*). The model prior *p*(*M*) is set by the researcher and evaluating the likelihood *p*(***y***∣***ψ***,*M*) for a specific model *M*_*k*_ is straightforward since the model-specific parameter vector ***𝜃***_*k*_ can be obtained from ***ψ*** using the function *g*_*k*_. The prior *p*(***ψ***∣*M*) is obtained by applying the change of variables theorem. Recall that $\boldsymbol {\psi } = g_{k}^{-1}(\boldsymbol {\xi }_{k})$ and ***ξ***_*k*_ = (***𝜃***_*k*_,***u***_*k*_). Furthermore, note that the prior *p*(***ξ***_*k*_∣*M*_*k*_) = *p*(***𝜃***_*k*_,***u***_*k*_∣*M*_*k*_) factorizes as *p*(***ξ***_*k*_∣*M*_*k*_) = *p*(***𝜃***_*k*_∣*M*_*k*_)*p*(***u***_*k*_∣***𝜃***_*k*_,*M*_*k*_).^22^ For clarity of what follows, let *f*_*k*_(***ξ***_*k*_) = *p*(***ξ***_*k*_∣*M*_*k*_). The implied prior on ***ψ*** under model *M*_*k*_ is then given by

11 $$ p(\boldsymbol{\psi} \mid M_{k}) = f_{k}\left( g_{k}(\boldsymbol{\psi})\right)  \left\lvert\frac{\partial g_{k}(\boldsymbol{\psi})}{\partial \boldsymbol{\psi}}\right\rvert, $$

where $\left \lvert \tfrac {\partial g_{k}(\boldsymbol {\psi })}{\partial \boldsymbol {\psi }}\right \rvert $ denotes the Jacobian determinant of the transformation. The Gibbs sampler can then be implemented by alternating between 1) drawing ***ψ*** from the full-conditional distribution *p*(***ψ***∣*M*,***y***) and 2) drawing *M* from the full-conditional distribution *p*(*M*∣***ψ***,***y***). Drawing ***ψ*** from *p*(***ψ***∣*M*,***y***) is accomplished as follows: one first draws ***𝜃***_*k*_ from the model-specific posterior *p*(***𝜃***_*k*_∣*M*_*k*_,***y***), then samples ***u***_*k*_ from *p*(***u***_*k*_∣***𝜃***_*k*_,*M*_*k*_), sets ***ξ***_*k*_ = (***𝜃***_*k*_,***u***_*k*_), and then computes $\boldsymbol {\psi } = g_{k}^{-1}(\boldsymbol {\xi }_{k})$. This means that one can conveniently post-process previously obtained model-specific posterior samples since a sample from *p*(***𝜃***_*k*_∣*M*_*k*_,***y***) can be obtained by selecting randomly a draw from stored model-specific MCMC output. The full-conditional distribution for the model indicator *M* is a categorical distribution, where *M*_*k*_ is sampled with probability

12 $$ p(M_{k} \mid \boldsymbol{\psi}, \boldsymbol{y}) = \frac{p(\boldsymbol{y} \mid \boldsymbol{\psi}, M_{k})  p(\boldsymbol{\psi} \mid M_{k})  p(M_{k})}{{\sum}_{j = 1}^{K} p(\boldsymbol{y} \mid \boldsymbol{\psi}, M_{j})  p(\boldsymbol{\psi} \mid M_{j})  p(M_{j})}. $$

We used the marginalized version of the Gibbs sampler described in section 2.3 of Barker and Link (2013). This marginalized version estimates the transition matrix $\boldsymbol {\Phi } = \left (\left \{\phi _{ij}\right \}\right )$, where *ϕ*_*i**j*_ = *p*(*M*^(*b*+ 1)^ = *M*_*j*_∣*M*^(*b*)^ = *M*_*i*_) and *M*^(*b*)^ denotes the sampled value for *M* at iteration *b* of the Gibbs sampler. The marginalized version does not require one to draw *M*; instead, one estimates **Φ** directly, one row at a time. The *i* th row of **Φ** is estimated by repeatedly 1) drawing ***ψ*** given model *M*_*i*_ from *p*(***ψ***∣*M*_*i*_,***y***) and 2) using the drawn ***ψ*** to compute *p*(*M*_*j*_∣***ψ***,***y***), *j* = 1, 2,…,*K*. A Rao-Blackwellized estimate of the *i* th row of **Φ** is then given by the average of the vector (*p*(*M*_1_∣***ψ***,***y***),*p*(*M*_2_∣***ψ***,***y***),…,*p*(*M*_*K*_∣***ψ***,***y***)) across draws from *p*(***ψ***∣*M*_*i*_,***y***). This process is repeated for all models *M*_*i*_, *i* = 1, 2,…,*K* to obtain an estimate of all rows of the transition matrix **Φ**. An estimate of the posterior model probabilities is then obtained by normalizing the left eigenvector of the estimated transition matrix corresponding to the eigenvalue 1. An advantage of this marginalized version is that instead of sampling models according to their posterior model probabilities, one can fix the number of samples for each model.

We applied this marginalized Gibbs sampler RJMCMC version to our single-participant example. The dimensionality of ***ψ*** was equal to the number of parameters of the full model. Under the full model, we simply set ***ψ*** = ***𝜃***_full_. Under the null model, there was one parameter less since *v*_true_ was fixed. Hence, the dimensionality of the auxiliary variable vector ***u***_*k*_ = *u* was one for the null model and we set ***ψ*** = (***𝜃***_null_,*u*). The auxiliary variable *u* was proposed from a distribution constructed based on a logspline fit (Kooperberg, 2016) to the posterior samples for *v*_true_ under the full model. Therefore, to relate the palette vector ***ψ*** to the model parameters (and the auxiliary variable for the null model), we used the identity mapping for both models (i.e., *g*_*k*_ was the identity function for both models); consequently, the Jacobian determinants of the transformations were equal to one.

## Footnotes

1 The numbers are based on a systematic literature review of published articles that fit the DDM and LBA to empirical data (Tran, 2018). A summary of the results is available at https://osf.io/ynwpa/.

2 A file that describes the content of the DMC tutorials and the different DMC functions is available from https://osf.io/kygr3/.

3 This release is available at https://osf.io/5yeh4/. It also contains a new tutorial that explicitly explains how to use the bridge sampling functionality in DMC (i.e., dmc_5_7_BayesFactors.R).

4 Other proposal distributions, such as a multivariate *t*-distribution, are also conceivable.

5 The warping procedure assumes that all parameters are allowed to range across the entire real line; if this is not the case, appropriate transformations can be applied to fulfill this requirement. Note that the resulting expressions need to be adjusted by the relevant Jacobian term.

6 Note that this choice is only optimal if the samples from the posterior distribution are independent, which is not the case when using MCMC methods. To account for this fact, we replace *N*_1_ when computing *s*_1_ and *s*_2_ by an effective sample size—the median effective sample size across all posterior components—obtained using the coda R package (Plummer, Best, Cowles, & Vines, 2006).

7 Note that in practice, we always run the iterative scheme in a more numerically stable way with respect to ${\hat {r}^{(t)} = \text {const} \times \hat {p}(\boldsymbol {y} \mid {\mathscr{M}})^{(t)}}$ (for details, see Gronau et al.,, 2017, Appendix B).

8 In principle, convergence is guaranteed (Meng & Wong, 1996), however, convergence may be so slow that it is infeasible to wait in practice.

9 It has been pointed out that $\hat {R}$ is not a perfect indicator of convergence in certain scenarios (e.g., Vehtari, Gelman, Simpson, Carpenter, & Bürkner, 2019). For a recent proposal of an improved $\hat {R}$, see Vehtari et al., (2019).

10 In its original form, bridge sampling has been proposed to estimate the Bayes factor directly. In line with, for instance, Overstall and Forster (2010), here we advocate a version that estimates one marginal likelihood at a time (see also, Meng & Schilling, 2002, section 1.3).

11 We thank Nathan Evans for attempting to apply simple Monte Carlo sampling to one of our hierarchical model comparison examples.

12 We provide R code for an exemplary hierarchical model (i.e., code for the *B*-model with data generated from the *B*-model using *n* = 20, *k* = 200) at https://osf.io/ynwpa/. The reason why we only provide code for one of the hierarchical examples is that (1) the data sets are simulated and one example is sufficient to show how to apply the method (the other examples are obtained via trivial changes to the code), (2) the corresponding files are *very* large. Files for the other examples are available upon request.

13 More fine-grained versions of Figs. 4 and 5 are available at https://osf.io/ynwpa/.

14 Note that in practice, very large log Bayes factor estimates as in this case (e.g., 880 − 920) yield the same conclusion independent of the exact number: overwhelming evidence for the favored model. However, when the estimated Bayes factor is closer to 1 (equivalently, log Bayes factor closer to 0), it is more important that the Bayes factor is estimated precisely as this may influence which model is favored (see, e.g., the single-participant example and the following example).

15 This model comparison may be regarded as artificial, however, the main goal of the example is to demonstrate that, even in the hierarchical setting, a Bayes factor of about 1 can be estimated precisely using Warp-III.

16 Another complication with approximate errors for separate marginal likelihood estimates is that it is not completely straightforward to derive an approximate error for the resulting Bayes factor estimate.

17 For multi-modal posterior distributions, both simple bridge sampling and Warp-III sampling may result in insufficient overlap between the posterior and proposal distribution, and should be used with caution.

18 Consequently, the code would need to be adjusted to allow for covariance matrix parameters or probability vector parameters where constraints apply jointly to several components.

19 As before, multivariate normality should hold for the appropriately transformed posterior distribution.
